# Mitotic and DNA Damage Response Proteins: Maintaining the Genome Stability and Working for the Common Good

**DOI:** 10.3389/fcell.2021.700162

**Published:** 2021-12-13

**Authors:** Fernando Luna-Maldonado, Marco A. Andonegui-Elguera, José Díaz-Chávez, Luis A. Herrera

**Affiliations:** ^1^ Unidad de Investigación Biomédica en Cáncer, Instituto de Investigaciones Biomédicas–Universidad Nacional Autónoma de México, Instituto Nacional de Cancerología, México City, Mexico; ^2^ Instituto Nacional de Medicina Genómica, Mexico City, Mexico

**Keywords:** DNA repair, mitosis, genomic stability, spindle assembly checkpoint (SAC), cancer, DNA damage response (DDR)

## Abstract

Cellular function is highly dependent on genomic stability, which is mainly ensured by two cellular mechanisms: the DNA damage response (DDR) and the Spindle Assembly Checkpoint (SAC). The former provides the repair of damaged DNA, and the latter ensures correct chromosome segregation. This review focuses on recently emerging data indicating that the SAC and the DDR proteins function together throughout the cell cycle, suggesting crosstalk between both checkpoints to maintain genome stability.

## Introduction

The eukaryotic cell has developed several surveillance mechanisms to ensure genome integrity throughout the cell cycle. Mainly, two mechanisms are responsible for maintaining genomic stability: the DNA Damage Response (DDR) and the Spindle Assembly Checkpoint (SAC).

There are exogenous and endogenous factors that continuously damage genomic DNA, such as radiation (UV and ionizing radiation) and free radicals, respectively. When DNA damage is generated, a series of reactions are initiated to identify and repair the damage; this mechanism is known as DNA Damage Response (DDR). The main function of the DDR is to recognize and repair DNA damage, generating a cell arrest to make way for repair, or if the damage cannot be repaired, the DDR induces cell death through apoptosis. If this mechanism fails, a tumorigenesis process can take place (Jackson and Bartek, 2009). As a canonical transduction signaling pathway, the DDR consists of a network of proteins that act as sensors, transducers, adaptors, and effectors in its regulation (Iliakis et al., 2003). A variety of DNA repair mechanisms characterizes the DDR. Together, they can repair the must of damage from the DNA (Hoeijmakers, 2009). The genomic maintenance system includes six principal multistep repair pathways, covering a specific subclass of DNA lesions (Giglia-Mari et al., 2011). Based on the type of damage, the DDR can block the progression through all major cell-cycle transitions, such as the G_1_/S and G_2_/M, and during the S phase (Zhou and Elledge, 2000). When the damage is extensive, and the DDR cannot repair it, cell death or apoptosis is triggered, thus protecting the organism from possible tumor development (Bernstein et al., 2002). Following DNA damage, the DDR sensor proteins (like PARP or DNA-PK) associate with the site of damage where they initiate downstream signaling to recruit damage transducers, which activate effectors to initiate the repair. Activation of transducers (such as CHK1 and CHK2) and effectors depends on the phosphorylation of two major kinases, ATM (Ataxia Telangiectasia Mutated) and ATR (Ataxia Telangiectasia and Rad3 related) (Lord and Ashworth, 2012). There are different types of DNA damage, particularly double-strand breaks (DSBs), are considered the most damaging and therefore have been most studied for their cellular implications and the development of drugs for cancer treatment (Terasawa et al., 2014). There are two types of mechanisms for repairing DSBs: Non-Homologous End-Joining (NHEJ) or Homologous Recombination (HR). The NHEJ simply brings two ends together, but bases may be lost or added (*indels*); this inaccurate process occurs mainly before replication without regard for homology. On the contrary, the HR provides high-fidelity because it uses the complementary or homologous sequence of the sister chromatid or homologous chromosome (Shrivastav et al., 2008).

On the other hand, the SAC participates in mitosis, controlling the transition from metaphase to anaphase, monitoring the union between microtubules and chromosome kinetochores, and the tension generated by this union to generate adequate chromosome segregation and thus ensure genomic stability (Wassmann and Benezra, 2001). The principal SAC molecular components involved BUB1, BUBR1, MAD1, MAD2, BUB3, CDC20, MPS1 and AURORA B. In the absence of microtubule-binding to kinetochores, the ubiquitin ligase activity of APC/C is inhibited by a complex called the Mitotic Checkpoint Complex (MCC), consisting of MAD3/BUBR1, MAD2, BUB1, BUB3 and CDC20. APC/C activity is regulated by binding its coactivator, CDC20, which is inhibited by the MCC. Thus, the interaction of CDC20 with MCC prevents APC/C activation and chromosome segregation. Once the binding and tension of microtubules with kinetochores have been generated, CDC20 dissociates from the MCC, activating APC/C. In this way, this complex can ubiquitinate its targets (i.e., BUB1 and Securin). APC/C ubiquitinates securin, favoring its degradation and releasing separase, generating the cleavage of the cohesins that maintain the union of the sister chromatids, allowing their segregation. Finally, APC/C ubiquitinates cyclin B, promoting the exit from mitosis (Musacchio and Hardwick, 2002; Yu, 2002; Jia et al., 2013).

Due to the phase of the cycle in which they were observed to participate, the DDR and the SAC were seen as two systems that acted independently. On the one hand, maintaining the genome’s integrity during the interphase and, on the other, ensuring adequate distribution of genetic material during mitosis, respectively. However, in recent years, many studies have determined crosstalk between the proteins of each pathway, maintaining genomic stability throughout the cell cycle, coordinating injury signals, inducing cell cycle arrest, and facilitating repair.

This review focuses on recent findings of the role of DDR proteins in mitosis and the SAC proteins regulating the response to damage to DNA.

## A Collaborative Work Between DNA Damage and Spindle Assembly Checkpoint Proteins

### DNA Damage Proteins in Mitosis

#### ATM

The kinase ATM (Ataxia Telangiectasia Mutated) is the core of the DNA damage signaling. It is activated by DNA damage, primarily for the response to double-strand breaks. ATM phosphorylates downstream targets that inhibit cell cycle progression, active the DNA damage repair or induce cell death through apoptosis (Matsuoka et al., 2007). The ATM response to DSBs depends on a trimeric complex constituted by MRE11, RAD50, and NBS1 (MRN) (Lee and Paull, 2005). This complex assembles at DSBs and holds the two ends together. ATM phosphorylates many proteins to initiate downstream signaling, including CHK2, 53BP1, the variant histone H2AX, and the MRN complex itself (Giglia-Mari et al., 2011; Sirbu and Cortez, 2013).

On fission yeast lacking the DNA damage checkpoint and using low doses of the DNA damaging agent methyl methanesulfonate (MMS), it was observed that cells are arrested in mitosis before anaphase. Furthermore, using cells whose chromosomes are unable to assemble to the kinetochore and therefore unable to generate arrest in mitosis, they observed that when cultured with the damaging agent, the cells were able to activate the SAC and generate mitotic arrest in contrast to untreated cells, concluding that this SAC-mediated arrest is independent of a functional kinetochore. However, this activation involves major SAC proteins such as Mad1, Mad2, Mad3, Bub1 and Bub3, inhibiting the activity of Cdc20 and Pds1 (Securin in higher organisms). Mainly, it has been determined that Tel1 (ATM homolog) and Mec1 (ATR homolog) inhibit anaphase initiation in the presence of DNA damage, on the one hand through the phosphorylation of Pds1, on the other hand, independently inhibits Pds1 turnover by inhibiting APC/Cdc20 through the activity of the SAC. Thus, both mechanisms converge in the modulation of Pds1; in addition, the authors open the possibility that Tel1 and Mec1 may participate in the activation of SAC when DNA damage is present. To a better understanding of how cells arrest anaphase, particularly in response to challenging DNA replication or damaged chromosomes, the authors propose on the one hand that the S-phase checkpoint prevents chromosome segregation through different signaling pathways via inhibition of mitotic CDK by Mec1/ATR and its effector kinases like Swe1/Wee1 and Rad53/Chk2. On the other hand, they propose that SAC can prevent chromosome segregation in the presence of DNA damage in cells where Tel1/ATM and Mec1/ATR are not functional through modulation of Pds1/securin. (Kim and Burke, 2008; Palou et al., 2017). Although these studies indicate that both pathways are interconnected, there is a need to explore more precisely whether this is occurring, opening the possibility of developing anti-tumor therapies that target both the SAC and the repair mechanisms in defective ATM/ATR cells so that the cells are selectively destroyed.

In a separate study, in HeLa mitotic cells and p53-deficient MEFs (mouse embryonic fibroblasts) treated with doxorubicin (to generate DNA damage), it was observed that ATM negatively regulate (promotes dephosphorylation) the Polo-like kinase 1 (PLK1) protein (a key molecule in mitotic progression) through the ATM/CHK1/PP2A pathway and that this dephosphorylation was independent of p53. PLK1 inactivation results in the accumulation of cells in the G2-like phase, blocking the cell division in response to mitotic DNA damage (Lee et al., 2010). However, although this work addresses the role of ATM and its involvement in PLK1 dephosphorylation during damage in mitosis, it remains to explore the mechanisms of regulation between PP2A and CHK1 and to determine in more detail their role in this pathway as well as study cell fate (apoptosis, death in G1, necrosis, genome instability) after cells suffer prolonged damage in mitosis.

**FIGURE 1:**
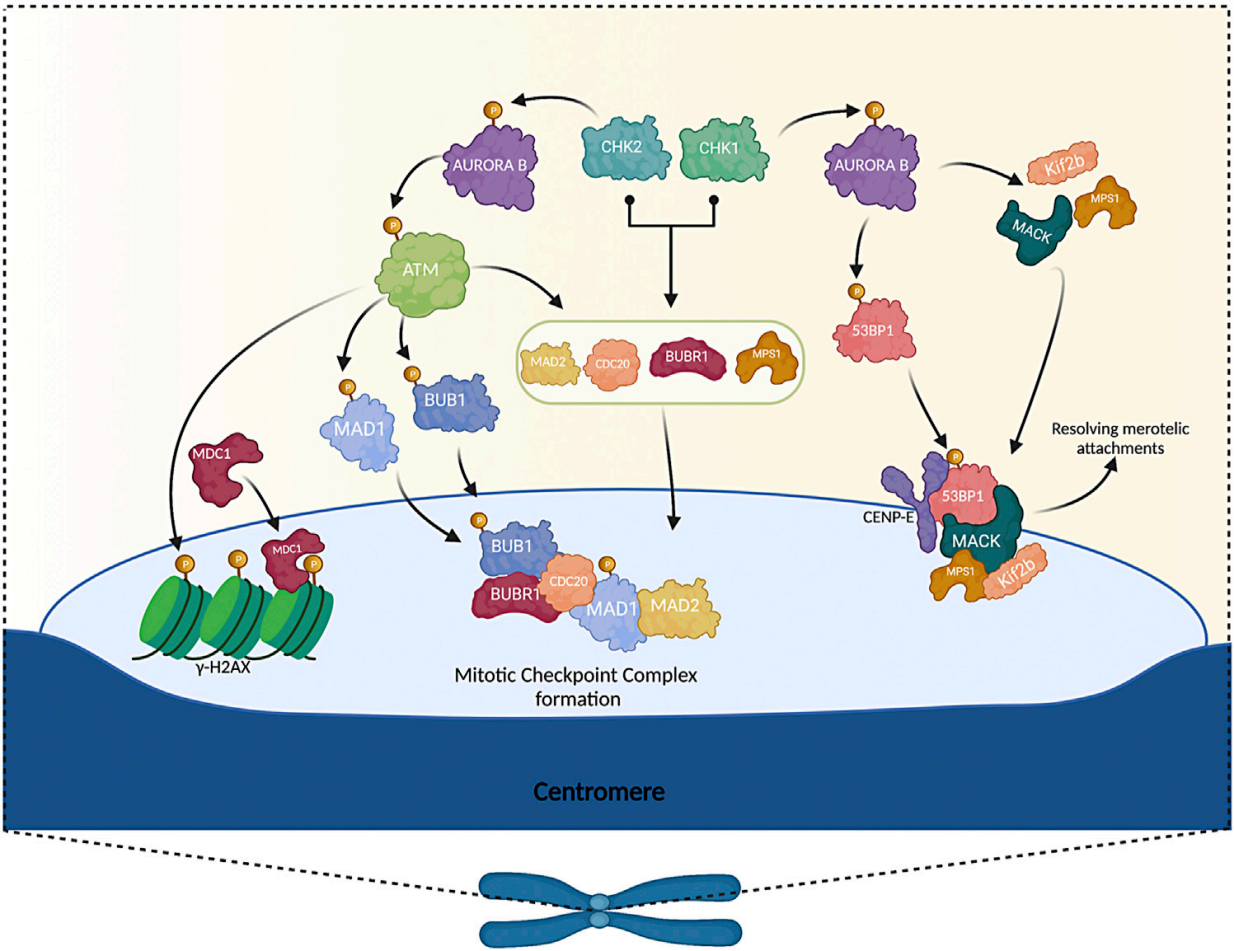
DNA damage response proteins and their role in the Spindle Assembly Checkpoint. ATM in the absence of DNA damage is activated dependent on AURORA B phosphorylation (Ser1403). ATM activated phosphorylates BUB1 (Ser314) and MAD1 (Ser214) to activate the SAC, endorsed the Mitotic Checkpoint Complex formation. Besides, ATM phosphorylates the histone H2AX (Ser139) at mitotic kinetochores promoting the MDC1 localization to the kinetochores, like MAD2 and CDC20 proteins, during SAC activation. CHK1 and CHK2 are capable of phosphorylated AURORA B (Ser331) in mitosis (at different stages), promoting his activation, and are capable of phosphorylated downstream targets, like ATM and 53BP1. CHK1 and CHK2 participate in the recruitment of MAD2, CDC20, BUBR1, and MPS1 to kinetochores. In addition, phosphorylation of AURORA B by CHK1 promotes the recruitment of MCAK, Kif2b, and MPS1 to collaborate in the resolution of merotelic attachments. AURORA B activated, promoted the localization of BUBR1 and MAD2 to the kinetochore during mitosis. Also, AURORA B phosphorylate 53BP1 (Ser1342) contributed to the recruitment of 53BP1 to kinetochores and colocalizing with CENP-E in the fibrous corona of kinetochores. Likewise, 53BP1 in kinetochores interacted with MACK, required for resolving merotelic attachments of chromosomes-microtubules during mitosis. Note: Created with BioRender.com.

Yang et al., report that in the absence of DNA damage, ATM can activate in mitosis. This activation depends on the phosphorylation of Serine 1403 by AURORA B, both *in vitro* and *in vivo* (Figure 1). It was also determined that autophosphorylation of ATM at Serine 1981 is important for its activation in mitosis and that mutation of ATM Serine 1403 leads to spindle checkpoint defects. They observed that ATM knockdown cells treated with nocodazole could not generate mitotic arrest and entered anaphase despite having misoriented or misaligned chromosomes in the metaphase plate. Finally, they discovered that ATM is capable of phosphorylates BUB1 at Serine 314 to activate the SAC (Yang et al., 2011); however, in a subsequent study, the authors determined that ATM phosphorylates BUB1 in mitosis in the presence of DNA damage at the same residue (see BUB1 section) (Yang et al., 2012). Further studies could investigate how AURORA B-mediated phosphorylation of ATM activates the enzyme in mitosis and whether ATM modulates or activates other mitotic targets in addition to those studied in this work.

In addition, it has been observed that in the absence of DNA damage, ATM phosphorylates MAD1 at Serine 214 (Figure 1), promoting MAD1 homodimerization and its heterodimerization with MAD2. In addition, it was demonstrated that mutant MAD1 (Ser214A) overexpression generates aneuploidy, indicating that this phosphorylation is necessary to avoid chromosomal instability (Yang C. et al., 2014). Because ATM activates BUB1 and MAD1 in mitosis in the absence of damage, it would be interesting to study what would happen to these and other SAC proteins if ATM is inhibited and damage is generated during this phase. Similarly, in mitosis, it is observed that ATM phosphorylates the histone variant H2AX at kinetochores (Ser139), promoting MDC1 recruitment at kinetochores. Besides, ATM as well as MDC1, are needed for an adequate localization of MAD2 and CDC20 during SAC activation (Eliezer et al., 2014) (Figure 1). These findings, together with those mentioned above, propose the following model: in the absence of DNA damage, ATM phosphorylates histone H2AX in kinetochores, recruiting MDC1, and the pre-complex formed by MAD2 and CDC20, which bind phosphorylated H2AX (γ-H2AX) and the proteins BUBR1 and BUB3 to form the Mitotic Checkpoint Complex (MCC) and to activate the SAC. However, in this study, it is not distinguished whether the role of ATM (and MDC1) in SAC activation is due to a function independent of DNA damage or whether the DDR activates the SAC.

These data indicate a possible function of ATM in mitosis, particularly in the function of SAC, and its purpose in the preservation of chromosomal and genomic stability (Boohaker and Xu, 2014).

#### CHK1

DDR promotes cellular cycle delay to promote DNA repair; this is carried out by checkpoint kinases 1 and 2 (CHK1 and CHK2), which are critical in repairing DNA damage and thus maintaining genomic integrity (Neizer-Ashun and Bhattacharya, 2021).

CHK1 plays an important role in response to DNA damage as an important cell cycle regulator. When DNA damage occurs, ATR partially activates CHK1 through phosphorylation at Serine 317 and 345, then CHK1 auto-phosphorylates at Serine 296, leading to its full activation. This autophosphorylation generates a binding site for 14-3-3 proteins, in addition to contributing to the physical interaction with the Cdc25C phosphatase, allowing CHK1 to phosphorylate and inhibit it. It also stimulates the activity of the WEE1 kinases that are responsible for phosphorylating the CDK/cyclin B complexes, generating an arrest in G2 and carrying out the DNA damage repair (Walworth et al., 1993; Flaggs et al., 1997; Sanchez et al., 1997).

Since its identification, CHK1 has been extensively studied, and its role in DDR has been determined. However, little was known about the role of CHK1 in mitosis and particularly in SAC. Since its identification, CHK1 has been extensively studied, and its role in DDR has been determined. However, little was known about the role of CHK1 in mitosis and particularly in the SAC. One of the first studies associating the participation of this protein in mitosis and SAC was carried out by Tang J. et al., who decreased CHK1 levels using RNAi in HeLa cells without DNA damage, observed that during metaphase, the chromosomes were not aligned in the metaphase plate and therefore at the time of chromosome segregation there were lagging chromosomes, due to the lack of attachment of the microtubules to the kinetochores. Furthermore, in CHK1-depleted cells, PLK1 and phospho-H3 (a mitosis marker) levels were increased compared to control cells, generating a premature entry into mitosis, and causing an arrest in this phase. In the same study, in thymidine-synchronized HeLa cells, it was observed that CHK1 could negatively regulate Polo-like kinase (PLK1) during mitosis, both in the presence and absence of DNA damage, also determined that CHK1 levels remained constant during the different phases of the cell cycle (Tang et al., 2006). However, it has been observed that synchronization with thymidine can generate DNA damage and consequently activate repair mechanisms (Darzynkiewicz et al., 2011). Therefore, it is necessary to check whether the observed constant CHK1 levels were not due to thymidine blockade, using a different method of cell synchronization that does not compromise DNA stability, e.g., starvation serum. Finally, the authors propose that by co-inhibiting CHK1 with MAD2 or BUBR1 (canonical components of SAC), CHK1 negatively regulates SAC (inactivating it) during the metaphase-anaphase transition, since it was observed that phospho-H3 levels are lower in cells with codepletion compared to CHK1-depleted cells (Tang et al., 2006).

In another study by Zachos et al., it was observed that CHK1 depletion in chicken DT40 (B-lymphoma cells) and CHK1 depletion by RNAi in human BE colon cancer cells generated chromosome missegregation and consequently chromosomal instability. The chromosome missegregation was also determined in CHK1-depleted human colon carcinoma HCT116 and human embryonic kidney HEK293 cells. To test the possible function of CHK1 on the SAC, spindle poisons, such as taxol and nocodazole (which stabilize microtubules by preventing their depolymerization and perturb the dynamics of microtubule formation, respectively) were used to avoid the assembly of the mitotic spindle with kinetochores, activating the SAC and delaying mitosis exit. They observed that CHK1-deficient cells treated with taxol could not generate mitotic arrest and that those treated with nocodazole were able to generate mitotic arrest. BUBR1 localization to kinetochores was decreased in the CHK1-deficient cells treated with taxol and during unperturbed mitosis; however, in CHK1-deficient cells treated with nocodazole, BUBR1 and MAD2 localized typically to kinetochores. They proposed that CHK1 participates in mitotic arrest in response to microtubule stabilization, but not when the arrest is through depolymerization. Likewise, in cells treated with taxol but not in cells treated with nocodazole, they determined that CHK1 is required for AURORA B kinase activity, although its localization to kinetochores is not CHK1-dependent (Zachos et al., 2007).

In a subsequent study by Petsalaki et al., it was observed that in undisturbed prometaphase or during spindle disruption by taxol but not with nocodazole (as in the study by Zachos et al., 2007), CHK1 could modulate AURORA B activity through Serine 331 phosphorylation. (Figure 1), prolonging the time of SAC activation and thus mitotic arrest, although this phosphorylation occurs in prophase, anaphase, and cytokinesis, even in cells treated with nocodazole, it is not indispensable for the localization of AURORA B to centromeres, nor for its autophosphorylation at Threonine 232 (activation loop) or its association with INCENP (a component of the chromosomal passenger complex, of which AURORA B is a component). It was even determined that in CHK1-depleted cells, Serine 331 was still phosphorylated in these phases, so it would be worthwhile to investigate which kinases could be involved in the phosphorylation of AURORA B in the absence of CHK1 and thus suggest that CHK1 is or is not indispensable for SAC function (Petsalaki et al., 2011). In addition, it has been shown that AURORA B and INCENP interact throughout the cell cycle, so it would be interesting to study whether or not CHK1 has a role during this interaction, independent of its function in DNA damage and SAC (Bolton et al., 2002).

Subsequently, in a study carried out by Petsalaki and Zachos using CHK1-depleted human BE cells and avian DT40 CHK1^−/−^ cells, they observed an increase in lagging chromosomes frequency merotelic attachments (microtubules of the same spindle pole join both kinetochores of the sister chromatids). To prolong metaphase, they treated the cells with MG132 (proteasome inhibitor). After release observed that in CHK1-depleted cells, the anaphases with merotelic junctions and lagging chromosomes did not decrease compared to control cells, suggesting that CHK1 is required to correct merotelic junctions before anaphase. The authors propose that CHK1 phosphorylates AURORA B at Serine 331 to promote, apart from modulation of AURORA B activation (as previously seen by Petsalaki et al., 2011) that: 1) MCAK and Kif2b (kinesins that serve to destabilize kinetochore-microtubule binding and correct mis-attachments) bind to kinetochores or centromeres, 2) favor phosphorylation of HEC1 at Serine 44 and 55 (modifications to promote kinetochore-microtubule detachment) and 3) that this phosphorylation of AURORA B by CHK1 promotes the recruitment of MPS1 to kinetochores. And that together, MPS1 and CHK1 generate the correction of merotelic junctions before anaphase (Figure 1); however, the mechanism by which they do it is uncertain, and it would be interesting how they manage to correct this junction since this type of error can evade detection by the SAC, generating missegregation, chromosome instability and consequently aneuploid cells (Petsalaki and Zachos, 2013).

In addition, in nocodazole-synchronized HeLa and BE cells and depleting CHK1 with a pool of siRNA, it was observed that cyclin B degradation took longer (2 h after arrest release) compared to control cells, indicating that CHK1 interferes with cyclin B degradation and consequently with mitosis exit. Furthermore, in HeLa cells where either CKH1 or AURORA B alone was deleted or a codepletion of both proteins was performed, in all three conditions, more than 50% of the cells were observed to have misaligned chromosomes, suggesting that CHK1, like AURORA B, may be involved in monitoring the binding of microtubules to chromosomes, in the tension generated by this binding, and possibly in the correction of merotelic junctions (Yang X. et al., 2014) (previously determined by Petsalaki and Zachos, 2013). Formerly studies had considered that CHK1 might have a role on some SAC proteins; in the Yang’s study, it was observed that in CHK1-depleted cells, BUBR1, MAD2, and CDC20 proteins were not localized in the unattached kinetochores, in addition, it was determined that the protein levels of MAD2 and CDC20 decreased, therefore, CHK1 is an important factor in the recruitment of CDC20, MAD2, and BUBR1 to kinetochores and the expression of CDC20 and MAD2 (Yang X. et al., 2014).

Yet, the mechanism by which CHK1 participates in the recruitment of these proteins to the kinetochore is not explored, and thus how CHK1 is involved in the modulation of CDC20 and MAD2 expression. Although it could be suggested that the delay of cyclin B degradation observed in this study could be related to the low levels of CDC20 protein due to the absence of CHK1 since the APC/C complex is activated in mitosis by the CDC20 cofactor and once activated, APC/C ubiquitinates cyclin B so that it is degraded *via* the proteasome, and the cell exits mitosis.

One of the aspects that had not been addressed in previous studies was to determine the function of CHK1 in mitosis and SAC in normal untransformed cells. Ju et al. discussed the role of CHK1 in early mouse embryo development through a specific CHK1 inhibitor (Rabusertib). They observed a high rate of lagging chromosomes and multipolar/unipolar spindles, resulting in defects in chromosome alignment at the first cleavage of embryos. Likewise, at the first cleavage, it was observed that AURORA B and BUBR1 were not recruited to kinetochores causing defects in kinetochore/microtubule binding compared to the control group. In parallel to exploring the functions of CHK1 in mitosis in early mouse embryos development, also explored its role in DDR in the same cells, since upon inhibition of CHK1, the γ-H2AX mark increases and RAD50 and RAD51 expression decreases, indicating that DNA damage is increased, as well as the mechanism to repair double-strand breaks is diminished. It was observed that ROS (reactive oxygen species) levels increase as a signal of DNA damage and that this increase triggers apoptosis mechanisms. Thus, these results demonstrate that CHK1 is an important protein in SAC function. Without its presence, the integrity of the segregation mechanism is compromised and its function in DDR, supporting the dual role of this protein in both mechanisms (Ju et al., 2020).

Although these studies indicate the possible role of CHK1 in mitosis and SAC, it is essential to continue to study CHK1 in this mechanism better to understand its function and impact on chromosome segregation. There are still exciting points to address; for example, what is the protein that modulates CHK1 activity in mitosis? How is CHK1 recruited to kinetochores? Does CHK1 have the same function in mitosis when there is DNA damage? Does CHK1 function as a scaffold protein to recruit other proteins to activate the SAC, or does it directly? And what is the role of CHK1 in mitosis?

#### CHK2

CHK2 is an important transducer of DNA damage signaling. In response to DNA damage, ATM phosphorylates and activates CHK2 at Threonine 68, allowing its binding to another CHK2 molecule, leading to its full activation by autophosphorylation at Threonine 383 and 387. Subsequently, CHK2 phosphorylates several key substrates, such as BRCA1 (Ser 988), favoring the initiation of DBS repair. Its substrates include phosphatases CDC25, p53, PML, E2F-1 and BRCA1. CHK2 also phosphorylates several CDC25 family proteins to arrest the cell cycle in the G1 phase and at the G_2_/M transition, preventing the cell from entering mitosis in the presence of DNA damage (Stolz et al., 2011).

**FIGURE 2:**
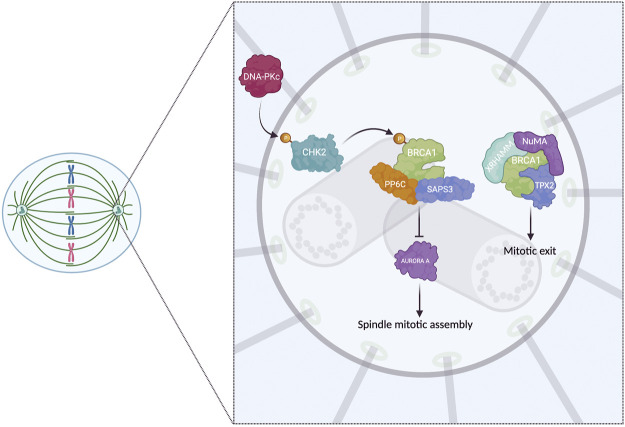
BRCA1 at centrosomes in the mitosis. In mitosis, BRCA1 colocalized with the centrosomes in the absence of DNA damage. CHK2 phosphorylated (Thr68) by DNA-PKcs, localizes at centrosomes and phosphorylates BRCA1 (Ser988), required to interact with PP6C and SAPS3 phosphatase, inhibiting AURORA A and promoting the assembly of the mitotic spindle. Further, it forms a complex with three spindle proteins, NuMA, TPX2, and XRHAMM, which ensures the mitotic exit. Finally, in the centromeres, BRCA1 interacts with y-tubulin (not shown). Note: Created with BioRender.com.

Its role in DDR has been well studied; however, in human colon cancer cells, it has been reported that in the absence of damage, BRCA1 is phosphorylated by CHK2 at Serine 988 at centrosomes; this phosphorylation is important for the PP6C-SAPS3 phosphatase complex to be recruited to kinetochores and also to interact with BRCA1, preventing, on the one hand, the binding of AURORA A to BRCA1 and on the other hand, inhibiting the activity of AURORA A, favoring a proper assembly of the mitotic spindle (Figure 2) promoting adequate chromosome segregation. Also, it was observed that the absence of CHK2 generates lagging chromosomes, increases chromosomal instability, and leads to the generation of aneuploid cells because the lack of CHK2 promotes the activity of AURORA A, resulting in an increase in the rate of microtubule assembly and leading to chromosome missegregation since AURORA A negatively regulates BRCA1 (trough phosphorylation of Serine 308) (Stolz et al., 2010; Ertych et al., 2016). However, it remains to be explored whether this phosphorylation of CHK2 on BRCA1 favors its ubiquitin ligase function in centrosomes and whether additional CHK2 target proteins exist during mitosis and how these proteins can regulate microtubules directly at the growing microtubule plus ends. On the other hand, under the same conditions and without DNA damage, CHK2 was phosphorylated at Threonine 68 and 387 during mitosis, and its kinase activity was increased during mitosis (Stolz et al., 2010).

In addition, using human colon cancer cells (HCT116) and osteosarcoma cells (U2OS), it was reported that CHK2 phosphorylated at Threonine 68 colocalized with centrosomes in mitotic cells (from late prophase until cytokinesis) and that this presence was PLK1-dependent. Furthermore, it was found that one of the targets of CHK2 is BRCA1 (in agreement with Stolz et al., 2010) and that the absence of CHK2 leads to errors in chromosome segregation and, consequently, chromosomal instability (Chouinard et al., 2013).

To know which protein phosphorylates CHK2 at Threonine 68 during mitosis, a study carried out by Shang et al., in HCT116 cells synchronized with nocodazole determined that it is DNA-PKcs that carries out this post-translational modification during mitosis and not ATM as occurs typically in interphase and the presence of damage. Therefore, it is proposed that CHK2 is part of a signaling pathway formed by BRCA1 and DNA-PKcs (DNA-PKcs-CHK2-BRCA1) to maintain genomic stability (Chouinard et al., 2013; Shang et al., 2014).

Finally, in human colon carcinoma BE cells, it is observed that CHK2 localizes to kinetochores in early prometaphase, when microtubules do not occupy most chromosomes. Furthermore, CHK2 stabilizes MPS1 protein levels through phosphorylation of Threonine 288. It has been observed that the catalytic activity of AURORA B is promoted by CHK2 phosphorylation at Serine 331 (CHK1 also phosphorylates this residue). Lastly, the presence of CHK2 in kinetochores promotes the recruitment of MPS1 and MAD2 to this site (Figure 1). It is important to note that CHK2 phosphorylates AURORA B at the beginning of mitosis (at early prometaphase) and CHK1 does so at prometaphase, so the activation of CHK1 and CHK2 and their activity on other proteins will depend on the phase of mitosis. Interestingly, the authors determined that phosphorylation of Threonine 68 in CHK2 was not indispensable for its activation in mitosis, contradicting what was reported by Shang et al., 2014. However, the discrepancy in both studies may be due to the localization of CHK2 during mitosis, since in this study they focus on CHK2 in kinetochores and Shang’s study, CHK2 is found in centrosomes, suggesting that the spatial regulation of CHK2 is important depending on its localization during this phase (Figure 1) (Saurin et al., 2011; Petsalaki and Zachos, 2014).

The main work on CHK2 in mitosis has focused on its involvement in centrosomes and its participation in mitotic spindle assembly; however, it is still necessary to investigate its role in kinetochores and thus in the SAC, since it is known to promote the recruitment of BUBR1 and MPS1 to these structures and it would also be interesting to investigate whether, like CHK1, CHK2 may play a role in correcting merotelic attachments that may occur during chromosome segregation.

#### 53BP1

53BP1 is a single-strand DNA binding protein and a critical effector of the NHEJ pathway DSB response. When DSB occurs, 53BP1 rapidly accumulates on the chromatin surrounding the break site and is recruited downstream of RNF8- and RNF168-dependent chromatin ubiquitylation (53BP1 recognizes and binds to the ubiquitinated Lys13 and Lys15 of H2A). This prevents end resection of the broken DNA strands and avoids the loss of genetic material at the damaged site (Fradet-Turcotte et al., 2013). 53BP1 also plays a crucial role in alleviating replication stress by recruiting NHEJ repair-associated proteins to stalled replication forks and stabilizing the DBS generated at these sites. However, this protein can also delay NHEJ repair at the G_2_/M transition by forming nuclear bodies on the strand breaks inflicted to alleviate replication stress. These structures enable timely segregation of the replicated chromosomes even in the presence of DSB, delaying their repair until the beginning of the G1 phase (Lukas et al., 2011).

The first evidence associated with 53BP1 and his direct role in the mitosis, mainly at the SAC, was observed by Jullien et al., they found in HeLa cells and NIH3T3 cells (embryonic mouse fibroblast) in the absence of DNA damage that 53BP1 is capable of colocalized with CENP-E in the kinetochore fibrous corona and with and with CENP-B during the prophase and prometaphase onset. In interphase cells, no colocalization of 53BP1 with centromeres/kinetochores was detected, indicating that this association occurs only in mitosis. Interestingly, chromosomes that were not aligned to the metaphase plate showed a higher 53BP1 signal than those already aligned; in metaphase, this signal was maintained at a lower intensity, which decreased until no signal was observed in mid-anaphase. Furthermore, 53BP1 is hyperphosphorylated in colcemid (inhibits mitotic spindle formation during mitosis) treated mitotic cells, suggesting that, like SAC proteins, 53BP1 is also a substrate for mitosis kinases (Jullien et al., 2002). Although these results indicate that 53BP1 may have a role in SAC and chromosome segregation, they do not delve beyond the localization of 53BP1 with kinetochores/centromeres.

Successively, in HeLa cells, it was reported that AURORA B is capable of phosphorylated 53BP1 at Serine 1342, contributing to their recruitment to the kinetochores (Figure 1). At the same time, it was found that 53BP1 is distributed in the kinetochores of chromosomes with merotelic attachments and is colocalized with the ACA (anti-centromere antibody) and HEC1 markers (both are markers of attached kinetochore to microtubules). When siRNAs or a 53BP1-S1342A mutant depleted 53BP1 was expressed, a significant number of lagging chromosomes in anaphase and the formation of metaphase bridges were observed. Furthermore, through mass spectroscopy screening and co-immunoprecipitation in mitotic cell extracts, it was identified that 53BP1 interacted with the mitotic-centromere-associated-kinase (MCAK) (Figure 1), suggesting that it is required for resolving merotelic attachments during mitosis. Therefore, in human mitotic cells, 53BP1 contributes to preventing aneuploidy by correcting spontaneous errors and merotelic attachments (Wang et al., 2015). However, it would be interesting to deepen how it participates in this mechanism like CHK1 could be another protein involved in the binding correction between microtubules and kinetochores to avoid chromosome missegregation.

Finally, another study observed that 53BP1 interacts with the APC/C co-activators, CDC20 and CDH1 proteins, through its tBRCT domain (during interphase) and KEN boxes (only during mitosis), with 53BP1 being an APC/C substrate at the beginning of mitosis. 53BP1 contributes to the inhibition of APC/C during the interphase, allowing the transition from S to G2 phase. Once the initiation of mitosis is reached, 53BP1 is ubiquitinated and degraded to allow the progression of mitosis, showing a reciprocal regulation between 53BP1 and APC/C. Furthermore, highly aneuploid tumors develop in 53BP1 knockout mice, supporting previous studies in which 53BP1 was associated with preventing aneuploidies (Kucharski et al., 2017). However, 53BP1 is not an essential protein for mitosis progression since it has been reported that 53BP1 knockouts animals are viable. Still, it could be an attractive therapeutic target since it has been observed that spindle poisons in 53BP1 knockdown cells can be lethal, and on the contrary, the use of APC/C complex inhibitors has been a way of killing tumor cells. Therefore, a combined therapy in tumors with low 53BP1 expression may result in successful treatment.

Although the previous studies associate 53BP1 with the SAC, more specific studies are still needed to help elucidate the mechanism by which 53BP1 could play a role in this phase of the cell cycle; whether or not it supports resolving merotelic attachments as well as if it has a direct function on SAC proteins.

#### BRCA1

BRCA1 and BRCA2 are essential proteins in maintaining genomic stability, participating mainly in the DDR through the HR pathway. Both proteins are considered tumor suppressor genes and are associated with susceptibility in breast and ovarian cancer (Miki et al., 1994).

In DDR, BRCA1 interacts with CtIP protein and the MRN complex (Mre11/RAD50/Nbs1) and participates in the dephosphorylation of 53BP1 to activate HR instead of NHEJ. In addition, BRCA1 functions as a scaffold for BRCA2 to be recruited to the repair sites. It has been observed that BRCA1 also has a role in repair by the NHEJ pathway, interacting with canonical proteins of this pathway, such as Ku80, participating in its stabilization at double-strand breaks (Venkitaraman, 2014; Gorodetska et al., 2019).

In COS-7 cells (simian virus 40-transformed monkey kidney) and through BRCA1-specific antibodies, it was determined that BRCA1 colocalized with the centrosomes in unperturbed mitosis. The signal was observed from prometaphase to the beginning of anaphase, and the signal was diminished in centrosomes when cells were in late anaphase and telophase. The co-localization of BRCA1 with the centrosomes was observed in human breast epithelial cells (BE46, E6/BE46, and 184A1), human embryonic kidney cells (HEK293), and breast cancer cells (MCF7). Besides, mitotic centrosomes were isolated from COS-7, and MCF7 cells, and also the presence of BRCA1 in mitotic centrosomes was also determined. Furthermore, an interaction between these two proteins was demonstrated through a co-immunoprecipitation of BRCA1 and γ-tubulin (a key component of centrosomes, responsible for nucleation of microtubules). However, it was not explored whether BRCA1 functions in mitotic centrosomes, although this would be one of the first pieces of evidence showing that BRCA1 is found in mitosis (Hsu and White, 1998).

Subsequently, Joukov et al. observed in *Xenopus* egg extracts and HeLa cells that the E3 ubiquitin ligase BRCA1/BARD1 (BRCA1-associated RING domain protein 1) heterodimer is involved in the recruitment of TPX2 protein to spindle poles through ubiquitination of TPX2, XRHAMM, and NuMA (involved in spindle-pole assembly) and of the negative modulation of XRHAMM, promoting the mitotic spindle-pole assembly. In *Xenopus* egg extracts, BRCA1/BARD1 was observed to interact with TPX2, NuMA, and XRHAMM. In human cell extracts, the complex interacts with NuMA, indicating that BRCA1/BARD1 is important in forming the spindle-poles assembly. Furthermore, in BRCA1-deficient cells, mislocalization of TPX2 to centrosomes was observed, resulting in mislocalization of AURORA A, the latter being important in TPX2 and BRCA1 phosphorylation. Finally, knowing that TPX2 and NuMA are targets of the Ran-GTP pathway and that multiple nuclei and micronuclei were observed in BRCA1/BARD1-deficient cells, due to the abnormal amplification of centrosomes in these cells, it can be suggested that BRCA1/BARD1 are involved in the correct function and formation of the mitotic spindle (Figure 2). However, it is unknown which mechanism BRCA1/BARD1 could be orchestrating this complex mechanism in centrosomes. Further studies are lacking to help elucidate the involvement of BRAC1 in mitotic spindle formation, particularly his role in centrosomes (Joukov et al., 2006).

Subsequently, in HCT116, it was determined that in the absence of DNA damage, AURORA A phosphorylates and inactivates BRCA1 (Serine 308), leading to chromosomal missegregation and chromosomal instability; however, this inactivation of BRCA1 is prevented by phosphorylation by CHK2 (Serine 988), leading to recruitment of the SAPS3-PP6C complex and preventing AURORA A from inactivating BRCA1 during mitosis (see CHK2 section) (Ertych et al., 2016).

To determine whether loss of BRCA1 would mimic the mitotic defects seen in CHK2-deficient cells, BRCA1-targeted shRNAs were used, observed abnormal mitotic spindle assembly and consequently delayed mitosis, restored by BRCA1 re-expression. In addition to the mitotic spindle, monopolar spindles were also detected when treated with monastrol (a kinesin-5 inhibitor), lagging chromosomes, and consequently chromosomal instability (Stolz et al., 2010).

The determination of this pathway formed by two tumor suppressor genes, such as BRCA1 and CHK2, is of vital importance since the loss of one or both can alter the assembly of the mitotic spindle, generating lagging chromosomes and consequently chromosome missegregation, leading to chromosomal instability and favoring the mechanisms associated with the development of carcinogenesis, It is, therefore, necessary to further investigate the role of these proteins, both in response to DNA damage and during mitosis.

#### BRCA2

Like BRCA1, BRCA2 is critical in maintaining genomic stability through its role in DDR. BRCA2 has been shown to participate in the recruitment of RAD51 and promote the displacement of RPA at sites of DNA damage, favoring HR repair. Besides, BRCA2 has an essential role in genome maintenance under conditions of replicative stress through the stabilization of RAD51 onto DNA and keeps the nuclease MRE11 inhibited, preventing chromosomal aberrations during replication stalling. Clinically, mutations in BRCA2 have been associated with predisposition to the development of breast, ovarian, and prostate cancer, among others (Wooster et al., 1995; Gorodetska et al., 2019).

In murine embryo fibroblasts (MEF) and HeLa cells targeted BRCA2 or using siRNA, cytokinesis was delayed, and some cells even failed to divide. In addition, myosin II has typically concentrated at the furrow formation dislocated in more than 50% of the cells and is undetectable at each cell edge. Moreover, it was observed that BRCA2 colocalizes with AURORA B in cytokinesis, particularly during elongation. Both proteins accumulate in the midbody during late cleavage and abscission. However, although BRCA2 may be present and regulate some processes during cytokinesis, it is not an essential component of the machinery for cell separation, such as INCENP, AURORA B and SURVIVIN, since, in CHK2-deficient cells, some cells were delayed in the division but mainly were able to carry out cell division (Daniels et al., 2004).

As in Daniels et al., 2004 study, Jonsdottir et al. observed prolonged cytokinesis in primary human fibroblasts (carrying a heterozygous mutation in BRCA2 gene, BRCA2^+/−^) compared to control cells (BRCA2^+/+^). Likewise, immunofluorescence showed that BRCA2 localized to the midbody in cytokinesis; however, in contrast to Daniels et al., BRCA2 did not colocalize with AURORA B (Jonsdottir et al., 2009) this discrepancy in whether BRCA2 colocalized with AURORA B or not may have been due to the specificity of the antibodies used in each study, although despite observing BRCA2 in cytokinesis and observing a delay in its completion in CHK2-deficient cells, neither of the two studies propose a mechanism by which CHK2 could be involved.

Subsequently, Mondal et al. studied more specifically the localization of BRCA2 in HeLa cells without DNA damage. Interestingly, BRCA2 localized throughout mitosis in centrosomes, in the spindle midzone during telophase, and in the midbody during abscission and cytokinesis. Using 293T cells (human embryonic kidney), they determined by immunoprecipitation that BRCA2 interacts with AURORA B, PRC1, and CEP55, which are involved in the completion of cytokinesis. In addition, they decided that BRCA2 recruitment to the central spindle and midbody is dependent on interaction with the protein FILAMIN A (a component of actomyosin complexes), involving FILAMIN A-dependent BRCA2 recruitment to these structures. On the contrary, they determined that BRCA2 is required for the recruitment of Alix and Tsg10 to the midbody. In turn, the ESCRT (endosomal sorting complex needed for transport) complex is recruited through the formation of the complex formed by CEP55-Alix and CEP55-Tsg10. That is, BRC2A plays an essential role in the assembly and signaling of components necessary for cytokinesis and cell division (Mondal et al., 2012).

Nevertheless, Lekomtsev et al. reported that BRCA2 is not necessary for the conclusion of cytokinesis in human cells. They observed that MgcRac depletion resulted in the accumulation of binucleated and multinucleated cells compared to BRCA2 depleted cells. Furthermore, by time-lapse in transfected cells with MgcRac siRNAs, binucleated cells were observed to form after the exit from mitosis, due to a failure in cleavage furrow; in contrast to cells with the BRCA2 siRNA duplexes, the cells generated the cleavage furrow after anaphase onset (Lekomtsev et al., 2010).

However, whether or not the observed defects in mitosis are due to the loss of BRCA2 was studied by Feng W. and Jasin M. In MCF10A cells (with a relatively stable genome) and using CRISPR-Cas9 mediated gene targeting towards BRCA2 (deleting exons 3 and 4, to generate a premature stop codon, preventing protein translation) they observed that BRCA2 deficiency led to replication stress in G2, causing subsequent aberrations in mitosis, such as chromosome missegregation and the formation of 53BP1-dependent nuclear bodies in the next G1 phase. In this phase, they observed that cell inviability was due to p53-independent apoptosis and senescence triggered by p53-mediated G1 arrest. This opens a new possibility of how BRCA2 function may impact mitosis (Feng and Jasin, 2017).

Moreover, in addition to its role in cytokinesis and cell division, the role of BRCA2 on some SAC proteins has been studied. In a study carried out in HeLa cells without DNA damage, immunoprecipitation determined that BRCA2 and BUBR1 interact during mitosis, particularly during prometaphase and at the outer kinetochore. Besides, it was also identified that the acetylation of Lysine 250 (K250) of BUBR1 and the presence of the protein in the kinetochores decrease in mouse embryonic fibroblasts with disrupted BRCA2 allele. This acetylation is known to be performed by PCAF to prevent degradation of BUBR1 by APC/C and is needed for accurate mitotic progression and SAC activity. Therefore, it has been suggested that BRCA2 works as a scaffold to enable the interaction between BUBR1/PCAF and thus facilitate the acetylation of BUBR1, contributing to the function of the SAC and avoiding chromosomal instability. However, whether this mechanism needs BRCA2-kinetochore localization is unclear, and by time-lapse microscopy, it was discovered that nocodazole treated BRCA2-deficient mouse embryonic fibroblasts exit mitosis faster than control cells (Daniels et al., 2004; Choi et al., 2012). That BRCA2 localizes with BUBR1 to kinetochores during prometaphase is interesting, contributing to the acetylation of BUBR1 preventing its ubiquitination by APC/C, generating a stronger wait signal carried out by the SAC until microtubules have not attached to kinetochores. It supports the idea that BRCA2-deficient cells are exiting mitosis faster, possibly because the SAC is weakened and unable to maintain the waiting signal for anaphase initiation since PCAF cannot acetylate BUBR1.

Despite the studies performed on BRCA2 and its participation in mitosis, the involvement of this protein in this phase of the cell cycle and the determination of whether BRCA2 is fundamental in the completion of cytokinesis is just beginning to be understood.

### Spindle Assembly Checkpoints Proteins in DNA Damage

#### BUB1

The *BUB* gene family (*Budding uninhibited By Benomyl*) was observed for the first time in experiments with *Saccharomyces cerevisiae* to identify key proteins in metaphase arrest (Li and Murray, 1991). In the vertebrates, BUB1 was found in the “unoccupied” kinetochores of chromosomes in the presence of agents that affect the mitotic spindle (Taylor and McKeon, 1997). BUB1 encodes a Serine/Threonine protein kinase and is a principal component of the SAC (Yu and Tang, 2004). In mitosis, it contributes to the kinetochore recruitment of essential components for the SAC functioning, such as SGO1 (Tang et al., 2004b), CENP-E, BUBR1, BUB3, and the dimer formed by MAD1-MAD2 (Sharp-Baker and Chen, 2001). BUBR1, MAD2, and BUB3 form the SAC complex, which keeps CDC20 inhibited and prevents it from activating the anaphase promoter complex (APC/C) (Peters, 2006), blocking the metaphase-anaphase transition. Furthermore, BUB1 inhibits CDC20 *in vitro* and *in vivo* through phosphorylation of the N-terminal domain (Tang et al., 2004a).

The first evidence that BUB1 could be involved in DDR was a large-scale proteomic analysis of proteins that could be phosphorylated by ATM and ATR in response to DNA damage. More than 700 proteins were phosphorylated by these proteins, including four SAC proteins, among them BUB1 (Matsuoka et al., 2007). Five years later, in HeLa cells, it was identified that ATM phosphorylates BUB1 at residue Serine 314 when DBS occurs by ionizing radiation (IR), and it was observed that BUB1 phosphorylated colocalized with the foci of γ-H2AX (a mark associated with DNA damage). Furthermore, by depleting BUB1 by siRNAs, the γ-H2AX signal was maintained for much longer than control cells. Besides, the phosphorylation of Threonine 121 of H2A performed by BUB1 (in order to the recruitment of SHUGOSHIN to the activation of SAC) is enhanced in response to IR, however, it’s well known that this mark is a mitotic-dependent event, independent of DNA damage (Kawashima et al., 2010). Therefore, indicated that this phosphorylation in BUB1 has other functions beyond mitosis (Yang et al., 2012). These findings suggest that BUB1 could have an important role in response to DNA damage.

**FIGURE 3:**
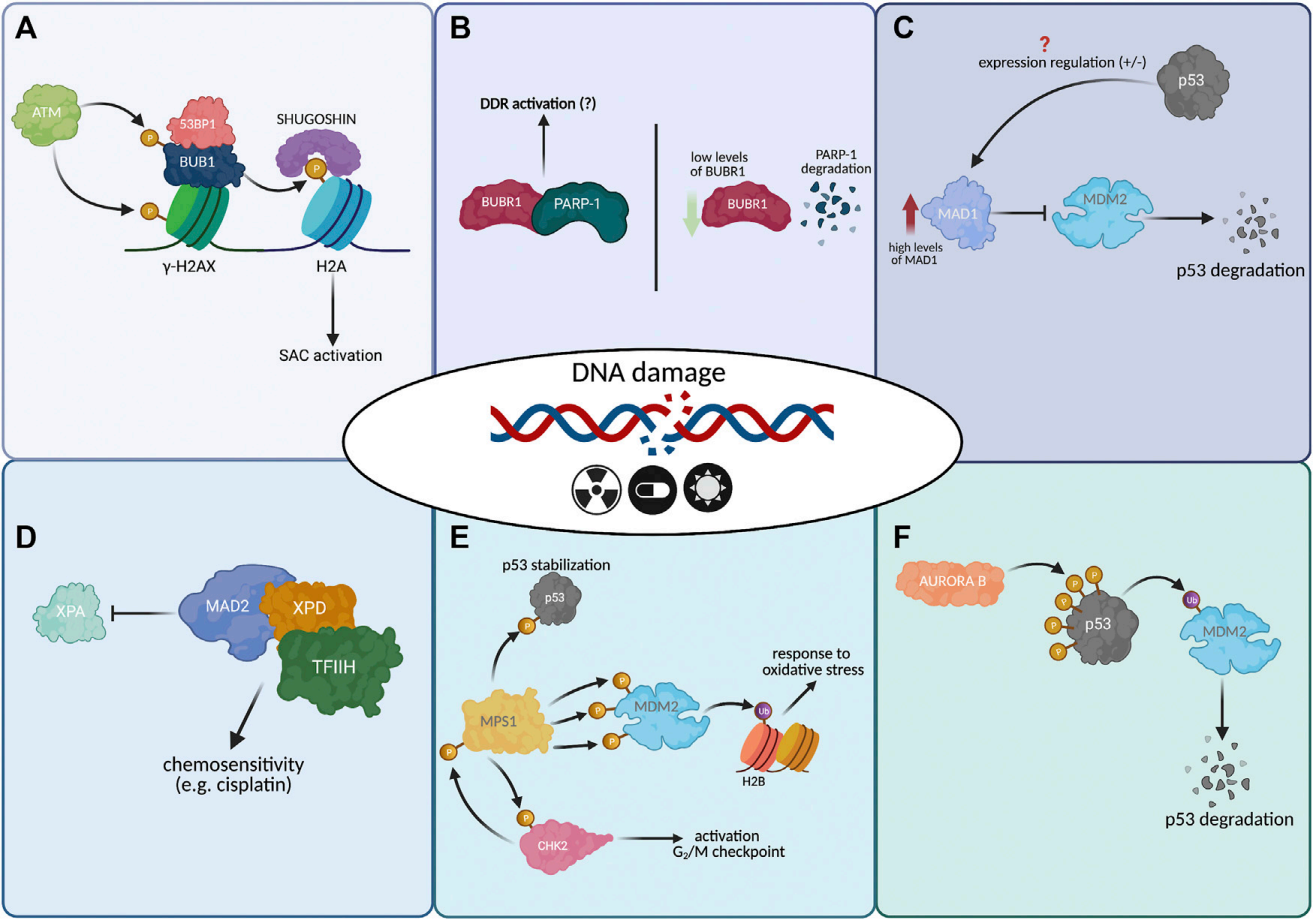
Mitotic proteins and their role in the DNA damage response (DDR). **(A)** In DSBs by ionizing radiation, ATM phosphorylates the histone variant H2AX (Ser139) and the BUB1 kinase (Ser314). This phosphorylation promotes the colocalization of BUB1 with the foci of the γ-H2AX, also of interacting with the DNA damage protein, 53BP1, associated with the NHEJ repair pathway. Further, BUB1 phosphorylates the H2A (Thr120), favoring the localization of SHUGOSHIN to the centromeres and activating the SAC (this last step is mitotic-dependent). **(B)** In response to DNA damage caused by UV-light or doxorubicin, BUBR1 can colocalize with PARP-1. Besides, in cells with low levels of BUBR1, PARP-1 cleavage is facilitated. However, it is unknown how BUBR1 could regulate PARP-1 and the DDR response. **(C)** Overexpression of MAD1 prevents the localization of MDM2 and promotes the degradation of p53 in the presence of DNA damage. Furthermore, p53 can regulate the expression of MAD1. However, it is not clear if this regulation on MAD1 inhibits its activity or represses it. **(D)** MAD2 interacts with XPD (a subunit of the transcription factor TFIIH), inhibiting the binding of XPA with XPD. This causes a decrease in the repair efficiency and increases sensitivity to drugs that damage DNA, e.g., cisplatin. **(E)** In response to DNA damage, MPS1 phosphorylates CHK2 (Thr68), promoting its activation, which in turn phosphorylates MPS1 (Thr288), promoting its stabilization and activating the G2/M checkpoint. MPS1 phosphorylates MDM2 (Thr4, Thr305, and Ser307), increasing the activity of MDM2 on histone H2B. It has been proposed that the ubiquitination of histone H2B participates in response to DNA damage by oxidative stress. MPS1 also binds to p53 and phosphorylates it at Thr8. This phosphorylation increases the stability of p53. This phenomenon has been proposed to be important in response to antimicrotubule drugs. **(F)** AURORA B interacts and phosphorylates p53 and promotes MDM2 ubiquitination and its degradation. The AURORA B expression reduces the transcription of p53 targets like p21 and BAX. AURORA B phosphorylated the residues: Serine 183, 215, and 269, Threonine 211 and 284; however, the relevance of each residue on p53 is unclear. Note: Created with BioRender.com.

Finally, the most recent study of the role of BUB1 in repair was the interaction by coimmunoprecipitation of BUB1 with the 53BP1 protein in HeLa and U2OS cells, an essential element in repair by Non-Homologous End-Joining (NHEJ) pathway, associating BUB1 with this repair path (Jessulat et al., 2015) (Figure 3A). However, the mechanism by which BUB1 participates in DDR and in which phase of the cell cycle it is participating has not been related in a particular way since NHEJ can act throughout the cell cycle, especially in interphase. Due to its kinase function, it could be inferred that it is involved in the recruitment and the phosphorylation of targets that participate in the DDR, either in the adapter or the effector proteins and in this pathway, making the response to DNA damage more efficient, therefore, studies are needed to elucidate its role in DDR.

#### BUBR1

Like BUB1, the *BUBR1* gene was identified in screens in *Saccharomyces cerevisiae* to identify genes and proteins that participated in mitotic arrest in the presence of spindle poisons (Li and Murray, 1991). Both genes are conserved in eukaryotes, and in conjunction, they participate in the function of the SAC (Chan et al., 1999). BUBR1 is a protein kinase, which is part of the mitotic checkpoint complex (MCC). Together with MAD2, BUB1, and BUB3, it contributes to the inhibition of the ubiquitin ligase APC/C, through the direct binding of CDC20 to complete arrest of mitotic progression (Fang, 2002).

The study that reported that BUBR1 has a role in response to DNA damage observed that cells heterozygous for BUBR1 failed to undergo significant mitotic arrest when DNA damage was generated using doxorubicin and UV. Furthermore, these same cells had low levels of γ-H2AX (a mark associated with the response to damage), and the expression of both p53 and p21 was significantly compromised before and after DNA damage. Moreover, BUBR1 colocalized and physically interacted with PARP-1, and in cells, BUBR1 deficiency facilitated the degradation of PARP-1. Due to the above and because p53 and PARP-1 are essential components in DDR checkpoint activation, it was proposed that BUBR1 has a role in the activation of this mechanism (Huber et al., 2004; Fang et al., 2006) (Figure 3B).

However, so far, it is unknown how BUBR1 regulates these proteins and modulates DDR; besides, there is no evidence in which BUBR1 is associated with the response to DNA damage during the interphase, limiting the participation of this protein to mitosis.

#### MAD1

MAD1 is a protein that participates in the SAC. It is located in the kinetochores from the beginning of mitosis to anaphase. MAD1 recruits MAD2 to the kinetochore, and this dimer promotes the conversion of other MAD2 molecules from the open to the closed conformation. The closed conformation of MAD2 binds to CDC20 and gives rise to Mitotic Checkpoint Complex (MCC), which inhibits APC/C until the beginning of anaphase (Musacchio and Salmon, 2007). Even though the main MAD1 activity has been studied during mitosis, its protein levels are constant throughout the cell cycle (Campbell et al., 2001). The transcriptional activity of its promoter is higher in G1 than at other points in the cell cycle (Iwanaga and Jeang, 2002) In interphase, it is located in the nuclear pores in union with MAD2 (Campbell et al., 2001).

MAD1 is overexpressed in malignant tumor-derived cell lines and breast tumor tissue samples. When MAD1 is overexpressed, it localizes in PML-NBs (ProMyelocytic Leukemia Nuclear Bodies). PML-NBs contain more than 100 proteins. Among these is the PML protein which participates in the p53 response to DNA damage. PML sequesters MDM2 in the nucleolus in response to DNA damage and allows the stabilization of p53. Overexpression of MAD1 prevents nucleolar MDM2 localization and promotes p53 degradation by binding to the PML protein. Then, MAD1 negatively regulates the response to DNA damage mediated by p53 (Figure 3C) (Wan et al., 2019). It will be significant to determine if the negative function of MAD1 on p53 occurs only during its overexpression and if these levels are comparable with those of tumors.

On the other hand, p53 can also regulate MAD1. In a global expression analysis, exogenous expression of p53 was shown to promote MAD1 expression in DLD1 cells (human colon cancer cell) (Polyak et al., 1997). However, Iwanaga et al. did not find an increase in MAD1 expression mediated by p53 wt, but there was an increase in MAD1 expression when the p53 281G mutant was transfected (Iwanaga and Jeang, 2002). Besides, p53 has also been described as a repressor of the MAD1 gene (Chun and Jin, 2003; Bansal et al., 2011). Therefore, the increase in MAD1 expression when the 281G mutant is expressed is not only due to the loss of repression of p53 wt, but of a gain of function of this mutant, as is the case in other examples such as the regulation of the gene hMMP-13 ((Sun et al., 2000; Gomes et al., 2021). The disparity between Polyak’s results and the further investigations may be due to the specific use in the first work of the DLD1 cell line, which possesses a specific p53 mutation (241 F), which may have some interaction with p53 wt that promotes MAD1 expression (Chun and Jin, 2003).

Therefore, with these findings, that p53 could regulate MAD1 in response to DNA damage. However, a study model must be established to determine the modulation of MAD1 expression by p53 and subsequently elucidate the mechanisms by which MAD1 could contribute to DDR. In addition, it would be interesting to investigate in which phase of the cell cycle this regulation is taking place, if it is in the presence of damage during interphase or if it is occurring during mitosis.

#### MAD2

Like MAD1, MAD2 is a key component of the SAC and binds to the kinetochore through MAD1. The binding of MAD1 to MAD2 causes a conformational change in MAD2 from an open to a closed configuration. The closed configuration interacts with more MAD2 molecules, converting them towards the closed configuration, which can bind to CDC20 and serve as the basis for MCC formation. Therefore, MAD2 is the effector of SAC by producing the APC/C major inhibitor (Lischetti and Nilsson, 2015). Like MAD1, MAD2 is expressed continuously during the cell cycle and has potential roles in interphase (Funk et al., 2016).

In interphase, MAD2 interacts with the XPD protein, a subunit of the transcription factor TFIIH that participates in nucleotide excision repair (NER), in the human embryonic kidney (HEK293) and cervical cancer cells (HeLa). A model has been proposed where MAD2 competes with XPA for binding to XPD. The decrease in the binding between XPD and XPA causes a reduction in the repair efficiency in cisplatin-treated cells, which generates damage to the DNA repaired by NER (Figure 3D). Therefore, MAD2 expression increases sensitivity to drugs that damage DNA (Fung et al., 2008). Consistent with this, it has been observed that in cell lines derived from oropharyngeal and gastric carcinoma, MAD2 levels are associated with cisplatin sensitivity, at higher levels, greater sensitivity (Cheung et al., 2005; Du et al., 2006). However, the role of MAD2 independent of mitosis must be well determined. In most studies, a mitotic arrest is not considered. The DNA damage can promote MAD2-dependent mitosis arrest that promotes cell death. Therefore, the different drug sensitivity may be related to the cell’s ability to repair damage or the efficiency in activating arrest in mitosis.

#### MPS1

MPS1 is a dual kinase that participates in the cell cycle in several mechanisms. It regulates the duplication of the centrosome and is part of the SAC mechanism. MPS1 has been demonstrated to be part of a feedback system with the CHK2 protein. MPS1 phosphorylates CHK2 at Threonine 68 in response to X-ray or UV light damage. Furthermore, this phosphorylation promotes both activation of CHK2 and the G_2_/M checkpoint by the same kinase. Wei et al. propose that this activity is independent of the CHK2 activation by ATM. This activity is not related to its role in SAC since the inhibition of MAD2 does not have a similar effect (Wei et al., 2005). On the other hand, CHK2 phosphorylates MPS1 at Threonine 288 and stabilizes the protein in X-ray-treated cells. Therefore, MPS1 participates in response to damage caused by ionizing radiation by activating CHK2 through a positive loop stabilizing MPS1 itself (Figure 3E). However, phosphorylation of MPS1 by CHK2 is important but not essential in the activation of the G_2_/M checkpoint (Yeh et al., 2009).

The interaction of MPS1 with the MDM2 and p53 proteins has also been observed. The suppressor gene p53 is one of the most frequently mutated genes in malignant tumors. p53 encodes for a transcription factor that promotes the expression of genes that arrest the cell cycle, promote DNA repair, and promote apoptosis under conditions of cellular stress. MDM2 is a ubiquitin ligase that ubiquitin to p53 and promotes its degradation (Levine, 2020; Borrero and El-Deiry, 2021). MPS1 phosphorylates MDM2 at the Threonine 4, Threonine 305, and Serine 307 residues, increasing the ubiquitin ligase activity of MDM2 on histone H2B. It has been proposed that the ubiquitination of histone H2B participates in response to DNA damage by oxidative stress (Khoronenkova et al., 2011) (Figure 3E). Therefore, MPS1 regulates the response to damage by phosphorylating MDM2 and promoting its ubiquitin ligase activity (Yu et al., 2016). MPS1 also binds to p53 and phosphorylates it at Threonine 18. This phosphorylation increases the stability of p53. This phenomenon has been proposed to be important in response to antimicrotubule drugs (Huang et al., 2009). Spindle poisons cause prolonged SAC-mediated mitotic arrest. Some cells die after being in mitosis for several hours. However, other cells are released from the arrest by a mechanism known as mitotic slippage (process by which a cell that is in prolonged mitosis exits this phase of the cell cycle due to a decrease in cyclin B below the threshold necessary to maintain the cell in mitosis) (Gascoigne and Taylor, 2008; Andonegui-Elguera et al., 2016). The cells released enter G1 and can be arrested in that phase, continue in the cell cycle, or die by apoptosis. It has been proposed that there is a p53-mediated tetraploidy checkpoint that arrests cells that undergo mitotic slippage in the subsequent G1 phase. Huang et al., found an interaction between MPS1 and p53 when treating cells with nocodazole or taxol. They propose that MPS1 regulates the tetraploidy checkpoint by stabilizing p53, promoting an arrest in the G1 phase after mitotic slippage. However, it has been observed that spindle poisons can cause DNA damage during mitotic slippage. Therefore, the MPS1-mediated p53 response could be related to direct DNA damage and not to the phenomenon of mitotic arrest induced by spindle poisons (Huang et al., 2009).

The results mentioned above propose that MPS1 actively participates in DDR. However, some questions must be clarified to understand the role of MPS1 in the interphase. First, it has been observed that MPS1 has a cytoplasmic localization during interphase (throughout the cytoplasm and focused on the centrosome and nuclear pores) (DOU et al., 2003; Liu and Winey, 2012) until the G_2_/M transition when is imported to the nucleus through two LXXLL motifs (Zhang et al., 2011). Besides, a putative Nuclear Export Signal (NES) has been found (Jia et al., 2015). In that respect, it will be important to determine if there is a basal location of MPS1 in the nucleus or imported during the response to cellular damage. Yeh et al., demonstrate a nuclear localization of MPS1 when it is phosphorylated at Threonine 288 in response to DNA damage (Yeh et al., 2009). Furthermore, MPS1 is a target of APC/C-CDC20 and APC/C-CDH1. Then, MPS1 protein levels during interphase are negatively regulated by APC/C (Cui et al., 2010). It will be important to assess whether APC/C plays a role in stabilizing MPS1 in response to cell damage. Although in one study, they did not find an association between stabilization and inhibition of the proteasome (Yeh et al., 2009).

### AURORA B

AURORA B is one of the multiple kinases involved in mitosis. It is part of the Chromosomal Passenger Complex (CPC), of which it is the catalytic component. AURORA B has different locations during mitosis associated with its specific function (Carmena et al., 2012). It is found in the chromosomes arms and the centromeric region during prophase, and towards anaphase, it relocates to the middle body (Wang et al., 2011; Kim et al., 2020). In the centromeric region, AURORA B negatively regulates the microtubule attachment to kinetochores. It participates in the formation of the contractile ring for cytokinesis in the middle body and the central spindle (Carmena et al., 2012).

In different tumors, overexpression of AURORA B has been observed associated with oncogenic potential. For this reason, different inhibitors of its kinase activity have been developed and are currently in clinical studies (Tang et al., 2015). It has been proposed that AURORA B may have a role independent of the regulation of mitosis. AURORA B interacts with p53 during mitosis and interphase (Carmena et al., 2012; Gully et al., 2012). In *vitro* studies, AURORA B has been demonstrated to be able to phosphorylate p53. Such phosphorylation promotes MDM2-mediated ubiquitination of p53 and its degradation. The p53 residues that can be phosphorylated by AURORA B are Serine 183, 215 and 269, Threonine 211 and 284; they are found in the DNA-binding domain of p53 (Figure 3F). However, each residue’s relevance in the AURORA B-mediated negative regulation of p53 is unclear (Wu et al., 2011a, 2011b; Gully et al., 2012; Jha et al., 2015). Thus, the overexpression of AURORA B could have an oncogenic role by negatively regulating p53 levels and decreasing the DNA damage response. Consistent with this, *in vitro* and *in vivo* studies have demonstrated that AURORA B expression reduces the transcription of p53 target genes such as p21 and BAX (important for promoting cell arrest and death) and decreases the efficiency of the response to DNA damage (González-Loyola et al., 2015). Although there is evidence of a possible relationship between AURORA B in DDR; there are still unanswered questions, especially regarding the role of AURORA B in DDR and tumorigenesis, since little is known about the regulation of AURORA B activity after DNA damage in G1.

## Conclusion

**FIGURE 4:**
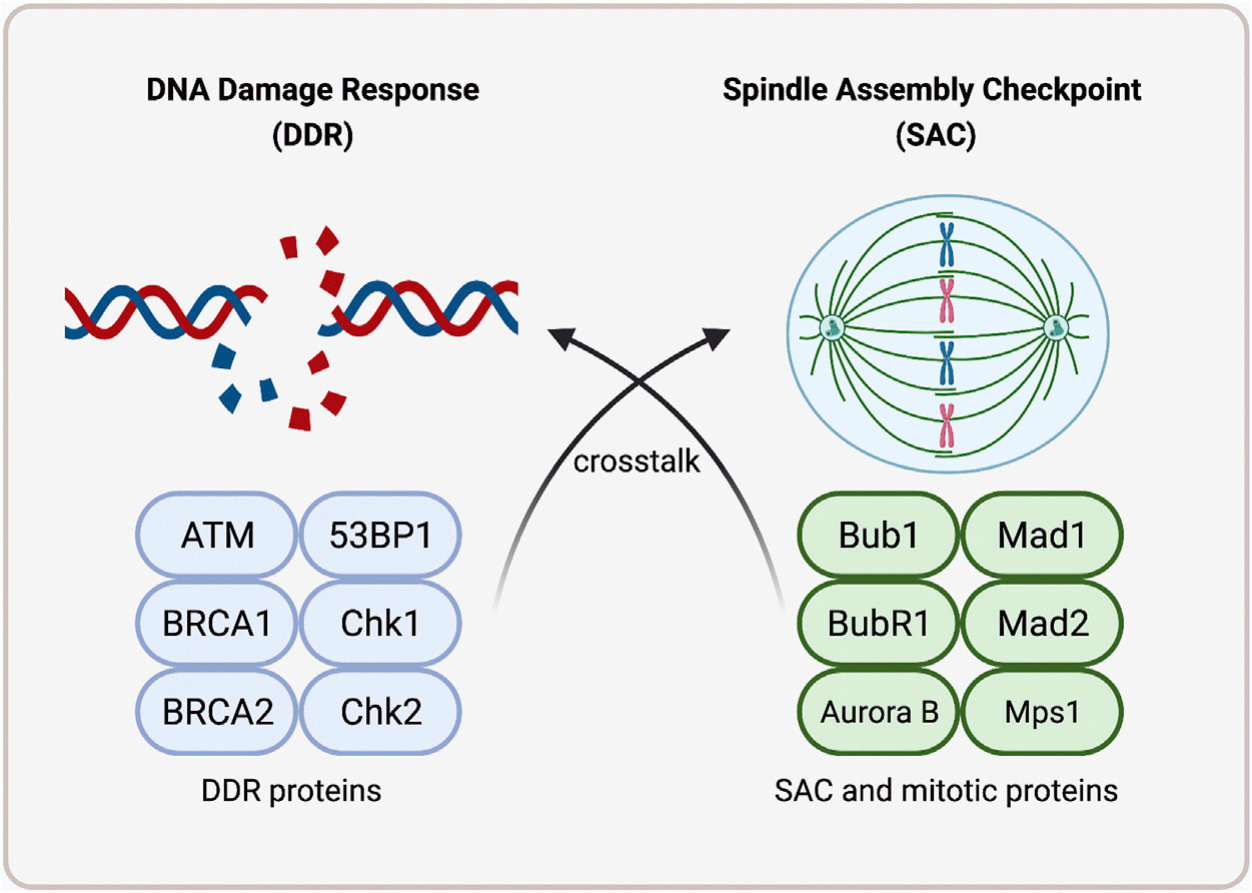
Crosstalk between the mechanisms of DNA Damage Response and the Spindle Assembly Checkpoint. Experimental evidence suggests that the two major cell cycle mechanisms, the DDR and the SAC, share common components, suggesting crosstalk between the two mechanisms in order to maintain the stability of the genome. Note: Created with BioRender.com.

One of the hallmarks of cancer proposed by Hanahan and Weinberg to understand the cell biology of this disease is genomic instability (Negrini et al., 2010; Hanahan and Weinberg, 2011). Chromosomal instability (CIN) is a type of genomic instability present in most types of cancer. The eukaryotic cell has several molecular mechanisms to avoid this type of instability and thus preserve the genome. The DNA Damage Response (DDR) and the Spindle Assembly Checkpoint (SAC) are two main mechanisms. Both mechanisms are involved during the cell cycle, coordinating DNA damage repair and ensuring proper chromosome segregation. Since both mechanisms participate at different cell cycle times, they were thought to be mechanisms that act independently. However, as recapitulated in this review, experimental evidence suggests that the two major cell cycle checkpoints share common components for maintaining genomic stability, which represents a significant step forward in this area, although these mechanisms remain to be further elucidated (Figure 4).

There is crosstalk, which has repercussions on how to study both phenomena, not as two separate and mutually exclusive pathways, and above all on the implications they could have on the development of cancer treatments. Most therapies to treat human cancers have been designed against specific molecular targets involved in a particular cellular pathway, favoring lower cytotoxicity in the treatment. However, it has been observed that cancer cells can survive without the function of the inhibited pathway, resulting in resistance to treatment, tumor re-growth, and consequently clinical relapse of the patient (Thompson et al., 2010).

In particular, the therapies have been designed to generate a considerable amount of DNA damage, and the cell cannot repair the damage, triggering cell death, or therapies that impact against the SAC mechanism or the mitosis (Tao, 2005). Both types of treatments have been developed independently without the notion that both mechanisms have elements in common that participate in both pathways. Thus, it is possible to design therapies targeting several signaling pathways or, in this case, essential mechanisms of the cell, such as the DDR and the SAC, to prevent the cell from developing resistance to the treatment. An alternative strategy would be to simultaneously promote chromosomal missegregation and deregulation of the DNA damage response through inhibition or disruption (co-targeting) of a well-established SAC or a component of the DDR, such as BUB1 or a component of the DDR, like BRCA2, respectively. Knowing both effects on the SAC and the role in the DDR, this therapy is expected to jointly impact these pathways and possibly the tumor response, preventing evasive or adaptive resistance of the cancer cell. Based on the work cited in this review and with future work to better understand the crosstalk between the two pathways, the development of better therapies for the treatment of human cancer is possible. However, our understanding of how the two pathways interact and mainly how they participate in maintaining genomic stability is still limited. Therefore, it is necessary to continue the work done so far to understand the molecular basis between DDR and SAC and its clinical implications in developing cancer treatments.
